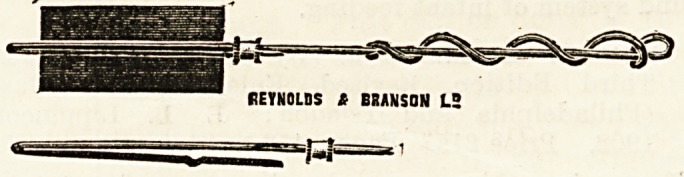# New Appliances and Things Medical

**Published:** 1904-01-30

**Authors:** 


### NEW APPLIANCES AND THINGS MEDICAL.

### UTERINE "FLAG MOP."

(As suggested by E. SOLLY, Esq., M.B.Lond., F.R.C.S.)

THIS instrument is designed to be of use as a substitute for Playfair's probe. The special advantages which it possesses over the latter are the ease with which bits of wool, lint, or gauze may be applied, and removed after use, while the device also prevents any chance of the swab accidentally slipping off. As will be seen from the illustration the material to be used is simply inserted between the two serrated halves of the split end of the instrument, and held in place by slipping up the small metal collar. If it be desired to protect the blunt extremity there is not the slightest difficulty in winding round the gauze or wool so as to effectually cover the end. There is a little projection at 2½ inches from the extremity, so that the appliance may be used as a uterine sound. The instrument, which is made of aluminium, can be readily bent into any curve that occasion may necessitate, although it is quite rigid enough for all practical requirements. We believe that anyone who gives the instrument a trial will find it to be of considerable value. The makers are Messrs. Reynolds and Branson, Leeds.

## Figures and Tables

**Figure f1:**